# Do dogs know what humans know? A study into pet dogs’ (*Canis* *familiaris*) ability to attribute knowledge to an unfamiliar person

**DOI:** 10.1007/s10071-025-02034-0

**Published:** 2025-12-12

**Authors:** Jori Noordenbos, Bonne Beerda, Hannah Layzell, Juliane Kaminski

**Affiliations:** 1 Behavioural Ecology Group, Wageningen University & Research, Wageningen, The Netherlands; 2 Centre for Comparative and Evolutionary Psychology, School of Psychology, Sport and Health Sciences, Portsmouth University, Portsmouth, UK

**Keywords:** Domestic dogs, Social cognition, Human-animal interaction, Competition, Knowledge attribution

## Abstract

Domestic dogs demonstrate sensitivity to human gaze and attentiveness, but evidence is mixed for their understanding of human knowledge, in the sense of understanding what a person has or has not seen. Here we tested whether pet dogs take a human competitor’s past visual access into account in a 3-choice food competition task. The dogs (*N* = 22) observed food being hidden under 2 of 3 cups, and witnessed how their human competitor saw only one baiting. When the human chose first, dogs were expected to avoid the cup she had seen baited; when dogs chose first, they were expected to choose randomly between baited cups, or favour the cup both had seen baited. However, the dogs chose randomly between the baited cups, regardless of who had first choice. The dogs did choose the cup only they had seen baited above chance only when the competitor chose first, suggesting a potential preference for this cup only in this situation, but they did not choose this cup more than the cup both had seen. Therefore, the current study provides no clear evidence that pet dogs infer others’ knowledge from past visual access. The combination of cognitive steps required to be successful in this task might have been too demanding for the dogs. Alternatively, the competitive setting used may not be as suitable for domestic dogs as it is for other species. Further research in ecologically relevant settings may clarify the extent and limits of dogs’ understanding of others’ knowledge.

**Supplementary Information:**

The online version contains supplementary material available at 10.1007/s10071-025-02034-0.

## Introduction

The awareness that others have different perspectives, desires and knowledge than oneself, or Theory of Mind (ToM), enables individuals to understand others and function in social groups (Frith, 2008). Understanding others’ desires and knowledge, which involves ‘role-taking’ abilities (Heyes, 1998), is considered to be cognitively more advanced than merely understanding another’s visual perspective. This perspective taking allows an individual to follow another’s gaze and understand that individual’s visual information, whereas the more advanced knowledge attribution is about grasping the other’s knowledge that logically results from the visual perspective (Heyes, 1998). In humans, ToM develops gradually with age, with kids of around 4 to 5 years old showing an understanding of knowledge in others (Carlson et al., 2013). Although ToM is a clear feature of mature human social cognition, the degree to which non-human species possess such social cognitive abilities is subject to ongoing scientific debate (Penn et al., 2008).

Dogs benefit from good social cognitive abilities to maneuver in their interspecific social environment and are highly attentive to humans. They are well adapted to read human body language and communicative cues without training, having even outperformed both our closest relative, the chimpanzee (Bräuer et al., 2006; Hare et al., 2000), and their closest relative, the wolf (Hare et al., 2000; Kubinyi et al., 2022; Virányi et al., 2008), indicating a role of domestication in the selection of interspecific social cognitive abilities (Bray et al., 2021). Subtle cues, like human head and eye direction, can direct dogs to a hidden food reward, and they selectively ignore a human’s gaze when they stare in the space above a hiding location (Soproni et al., 2001). Moreover, dogs seem to recognize intentional communicative signals by humans (Kaminski et al., 2012) and are also sensitive to their attentional states and visual perspectives (Gácsi et al., 2004; Virányi et al., 2004). For instance, dogs did not take forbidden food when a human was watching, but did so when they could take it undetected, e.g. when the person had his/her eyes closed, was distracted or turned around, or when the person’s vision was obstructed by a barrier (Bräuer et al., 2004; Call et al., 2003). Similarly, dogs used a person’s visual perspective on two toys to infer which toy an experimenter wanted, when this person commanded the dog to ‘fetch the toy’ (Kaminski et al., 2009). The toy behind the transparent barrier, which was visible to the experimenter, was retrieved more often than the toy behind the opaque barrier, but not so when the experimenter changed their position so they could see neither or both toys (Kaminski et al., 2009).

While dogs understand others’ current visual access, there is of yet not much evidence that dogs understand others’ knowledge that comes with past visual access. In contrast, chimpanzees, another social and intelligent species, do show such a sophisticated understanding of others’ past visual perspective and resultant knowledge in competitive paradigms with conspecifics. The subjects most often chose the food reward location that a competitor had not seen being baited, whilst choosing randomly between baited locations when the baiting was visible for both subject and competitor (Hare et al., 2001; Kaminski et al., 2008). So far, there is little evidence for this kind of knowledge attribution in dogs. In a second study by Kaminski et al., (2009), it was tested whether dogs would attribute knowledge to an experimenter, based on this person’s past visual access. Similar to the first paradigm, they positioned an experimenter and a dog opposite to each other, now separated by two opaque barriers. In view of the dog, the experimenter saw the hiding of a toy behind one of the two barriers, and left the room, so that only the dog saw how a second toy was placed behind the other barrier. Similar to the first study, the experimenter would command the dog to ‘fetch the toy’, but this time the dogs did not make a distinction in the retrieval of one toy or the other (Kaminski et al., 2009). This suggests that unlike chimpanzees, dogs may not be able to attribute knowledge to others. However, dogs did selectively follow the pointing gestures of a knowledgeable helper (knower), disregarding those of an ignorant (guesser), when locating a hidden food reward (Catala et al., 2017; Maginnity & Grace, 2014). The knower witnessed the hiding of the reward whereas the guesser was outside the room at the time of the hiding or looking in another direction. Seemingly, the dogs understood that only one person had the relevant knowledge based on what he/she saw earlier, but this interpretation is subject to scientific debate. Dogs may learn simple associative rules to solve complex tasks successfully, like focusing on the knower because this person had their eyes on the food reward the entire time. Nonetheless, Catala et al., (2017) did add a control condition in which both experimenters gazed in the same direction and dogs showed a preference for the knower, based solely on this person’s position and the visual access that could be inferred from that. This geometrical gaze following may indeed show a rudimentary form of knowledge attribution in dogs, and further studies, using different paradigms, are needed to confirm these findings.

There is not one study or paradigm that can conclusively demonstrate if dogs are capable of attributing knowledge to humans, but more research can provide indirect evidence and support a most likely interpretation of what dogs do and do not understand. Given dogs’ sensitivity to a human’s perspective and attentiveness, and their reliance on humans for resources, it seems plausible that dogs can understand what humans know, based on past visual access. Dogs failed to do so in the cooperative context of Kaminski et al., (2009), but this does not exclude that dogs show knowledge attribution in other situations, such as during competition. Dogs are a competitive species that form dominance relationships (Bradshaw et al., 2016; Cafazzo et al., 2010). Free ranging dogs (FRD) and dogs living in packs show extreme food competition, and lower ranking individuals will leave food when it is claimed by a more dominant individual (Cafazzo et al., 2010; Dale et al., 2017). For these lower ranking individuals, it would be beneficial to understand when a higher ranked individual does not know the location of food, so they can safely approach it. This was found for chimpanzees, where lower ranked individuals did not go to a hidden food reward if they saw a more dominant individual also observe the hiding (Hare et al., 2001). Kaminski et al., (2008) later showed that chimpanzees take conspecific knowledge on the location of food rewards into account in a competitive set-up, irrespective of the hierarchy between the individuals. Although pet dogs generally do not have to compete with other dogs, or with humans, to survive, there is no reason to assume that they lack the ability to engage in competitive situations. Pet dogs still compete with conspecifics for resources, such as toys, sleeping places and food rewards (Jacobs et al., 2018) and in the same way dogs may also compete with their owner. The finding that pet dogs will wait until their owner is not watching before they take a forbidden food reward (Call et al., 2003), shows their desire for food, even in the presence of humans. We therefore expect pet dogs to take the perspective, and possibly resultant knowledge, of a human into account when making choices, if this gets them food. The aim of this study was to find out whether dogs take the perspective and resultant knowledge of humans into account to maximise their own food outcome.

The paradigm used for the dogs in this study was modelled after a paradigm previously used and developed for chimpanzees by Kaminski et al., (2008), allowing also for a comparison between species. The dogs could earn food rewards by choosing a baited cup out of three cups, of which two held a food reward. They saw the baiting of both cups and observed that a human competitor saw only one baiting. During the other baiting, and the lifting of the empty cup, the vision of the competitor was blocked with opaque curtains. The cups would alternately be presented to both competitors, dog and human, who could make choices until both food rewards were gone. The first choice was always invisible to the opponent, using those same opaque curtains. This forced the dogs to consider the view of the competitor when she chose first, which was the case in half of the test conditions. In these conditions we assumed knowledge attribution if dogs chose the cup of which only they had seen the baiting, showing an understanding that the other baited cup had likely already been emptied by the human. In the other test conditions, when the dog chose first, we expected dogs to choose randomly between the 2 baited cups, since that would show an understanding that both cups still held food rewards. Another option, although less likely, would be that dogs would prefer the cup both had seen baited. This would show an understanding of what the human would choose in the next trial, maximizing food outcome for the dog. This tactic was seen in children and adult humans in the same paradigm, but not in chimpanzees (Kaminski et al., 2008). Control conditions were included to check if dogs followed the general course of events and did not make use of uncontrolled cues. If dogs are capable of taking the knowledge of an opponent into account in a competitive set-up such as the one modelled here, this would show further evidence of knowledge attribution in pet dogs. Understanding whether dogs can attribute knowledge to humans deepens our grasp of the social cognition of domesticated species, improves training and cooperation with dogs, and strengthens ethical approaches to dog welfare.

## Methods

### Subjects

Twenty-two privately owned pet dogs of various breeds participated in this study, with 13 males and 9 females ranging in age from 1 to 10 years old (S1 table). They were recruited from the database of the Dog Cognition Centre (DOCS) in Portsmouth. Twelve dogs had participated in earlier studies at the DOCS, but never on perspective taking or understanding knowledge in others. Owners of the dogs were asked not to feed their dog just before coming to the research facility. Water was available to the dogs at the research facility.

### Experimental procedure

Dogs were tested for understanding knowledge in humans in a 3-choice food competition task with a female human competitor. The dog subject saw 2 food rewards being hidden underneath 2 out of 3 cups, with the third cup remaining empty. The human competitor saw only one food reward being hidden, as witnessed by the dog. Both made choices alternately until both baited cups were chosen. The precise cup of first choice, including the experimenter lifting the cup and handing the food to the chooser, was always hidden for the other individual, but the rest of the choices were visible.

#### Set-up

**Fig. 1:**
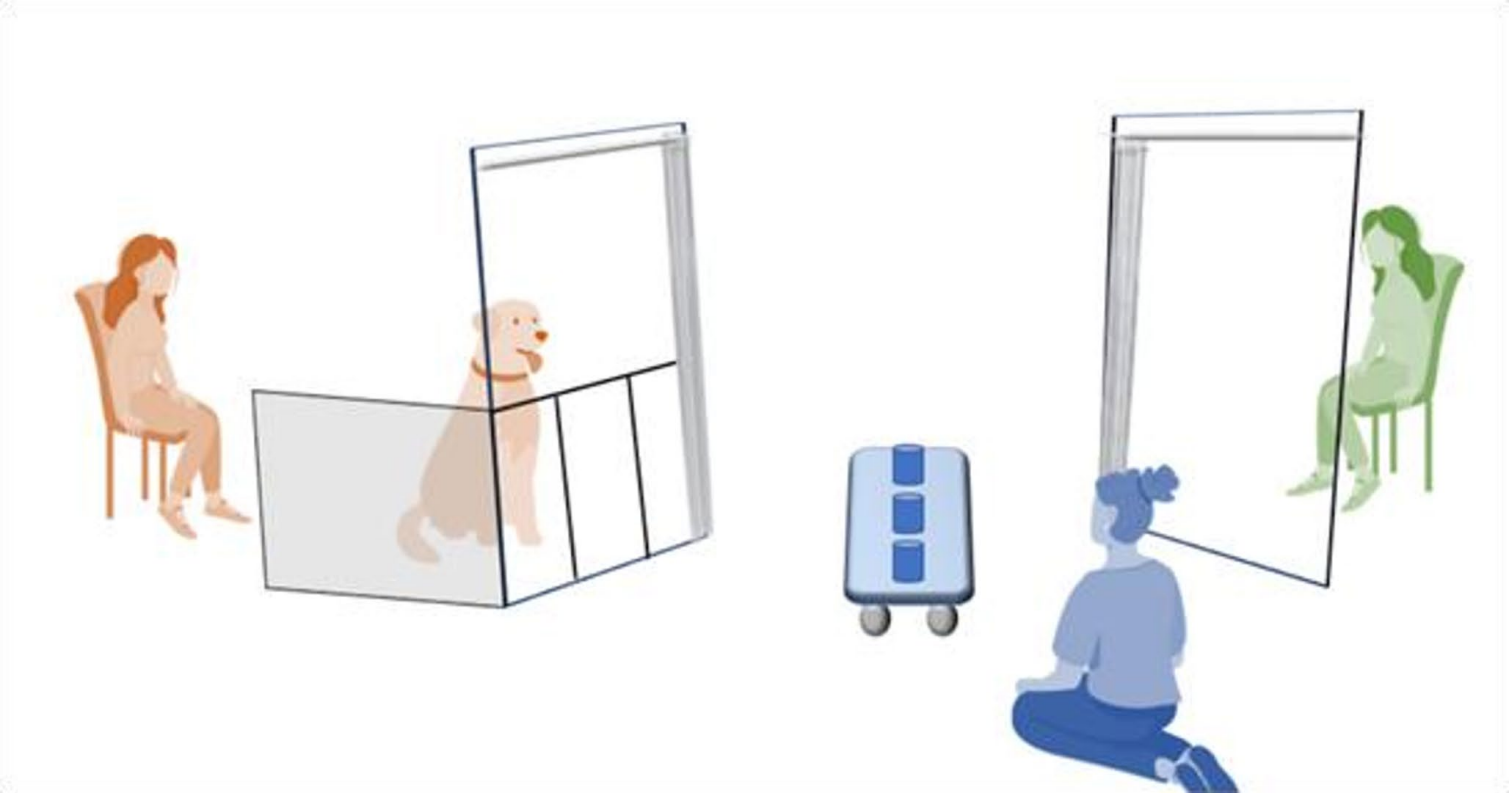
Schematic overview of the experimental set-up. The dog and its owner are on the left and the human competitor on the right. The experimenter sat behind the rolling board on the floor, to bait the cups, open and close the curtains and roll the board over to either the dog or competitor

Testing took place in a quiet room at the Dog Cognition Centre (DOCS) in Portsmouth. Two chairs were placed opposite each other, with 2 m in between (Fig. 1). The owner of the dog sat down on one chair with the dog in front of them. A female competitor who was unfamiliar to the dog sat on the opposite chair. An experimenter sat on the floor, at the midline of the set-up, equidistant from dog and human competitor, and behind a wheel board (80 × 25 cm) with three cups placed on it. The experimenter could roll the board to the side of either the dog or human competitor to allow it/her to choose a cup and receive the food reward under it. After choosing, the cup would be placed back on its original position, so that there were always 3 closed cups on the board. Opaque curtains in front of each chair allowed the experimenter to block the view of either the dog or human competitor as required (Fig. 1). During testing, dogs were off leash in their designated area, or on a loose leash if they were otherwise difficult to control. The dog’s designated area was in front of its owner and behind a fence that we constructed in between the curtain poles, consisting of a panel with 3 openings (22 cm wide and 65 cm in height), which aligned with the positions of the 3 cups. Dogs could put their head or paw through an opening to indicate a cup by touching it or staring at it, standing in the corresponding opening, making the choice for a specific cup easier to score. The cups were cleaned and subsequently rubbed with smelly kibbles before each session as to control for odour. The food rewards used during training and testing were high quality kibbles with salmon and trout, unless owners preferred to use their own small treats or food pieces. A GoPro camera filmed the dog’s choices from above.

#### Training phase

The dogs were trained step-wise for the task at hand, learning about the role of the competitor and how to indicate the cup of choice. Only dogs that passed a pre-test learning criterion entered the test phase. The curtains in front of the dog and the competitor stayed open during training and pre-testing. Dogs were first taught how to indicate a cup in four consecutive steps. First, one cup was placed on the board. The experimenter showed a food reward to the dog and put it visibly on top of the cup, before rolling the board over to the dog, allowing it to pick up and eat the treat. This was done for all 3 positions on the board, making the dog use all 3 panel openings. Owners were allowed to give the dog a release word and to verbally praise it when making a choice, but they were instructed not to interact or interfere otherwise.

When the dog managed to take treats from the top of the cup via the corresponding panel openings, the experimenter put the food reward underneath the cup, before rolling the board over to the dog. The dog received the reward from the experimenter when it touched the baited cup by either its nose or its paw, or when it intently stared at a specific cup while standing in the corresponding opening. If the dog chose incorrectly one or more times, this step was repeated until the dog got it right. In the third step, the number of cups was increased to two and at this point the experimenter lifted both cups one-by-one during the baiting. The cups were always lifted in the same order, with the cup closest to the experimenter first. Food was placed underneath only one of them. If dogs showed a preference for one of the positions, the treat was temporarily placed on top of the cups again. In the fourth step, the number of cups was increased to three, following the same baiting rules as during the third step. When the dog was correct 5 out of 6 times in a row, a short break was introduced before the pre-test began.

During the pre-test baiting, the experimenter placed food rewards under 2 of the 3 cups and lifted the third cup, showing the dog that nothing was hidden there. The human competitor also observed the baiting of two cups and the lifting of the third, and got to choose first in half of the pre-test trials. The experimenter rolled the board over to either the dog or the human competitor and the choices made were visible to both. Just as in the training, dogs chose a cup by touching it with either their nose or paw, or by staring at a cup while standing in the corresponding panel opening. The competitor always chose a baited cup, and did so by pointing to it. After a choice by the dog or the human, the experimenter would silently lift the chosen cup and give the food reward (if present) to the chooser. The chosen cup would afterwards be silently placed back again on the board. Subject and competitor were not allowed to pick up the treat themselves and the handling of the cups happened silently to avoid the opponents hearing each other choice in the later test trials. If the dog chose an empty cup, the cup was lifted without giving a reward and the board with the 3 cups was rolled over to the competitor to choose. The board was rolled back and forth between the 2 choosers until the 2 baited cups were emptied, to teach the dog about the actions of the human competitor and the consequences of choices for reward locations. There were maximal 2 training days with 4 pre-test sessions of 6 trials each, and dogs passed the pretest when they picked a baited cup in at least 5 out of 6 trials within one session. Which 2 cups held a food reward was randomized per trial. Twenty-four out of 42 dogs completed the pretest successfully, of which one dog was withdrawn by the owner and one was excluded for behaving unruly, leaving 22 test subjects. Dogs that dropped out during the pretest were mainly excluded for developing a strong side bias that could not be overcome within the 2 training days.

#### Testing phase

The testing phase included 48 test trials, evenly divided over 2 days, except for one dog that missed the second test day. The 2 test days were separated by 2 to 9 days, and each day included 2 sessions of 12 trials with a break in between the sessions. The procedures were similar as during the pre-tests, except for the use of curtains to temporarily block the cups from the view of the dog or competitor when needed, depending on the condition. In each condition, the dogs observed how food rewards were placed under 2 of 3 cups and how the third cup was lifted without being baited. Which cups were baited, and which was seen by the competitor was semi-randomized in such a way that the same set of cups was never baited two times in a row. What the competitor saw depended on the condition. There were two experimental conditions: **Competitor First choice** (Exp-CF) and **Subject First choice** (Exp-SF). In these conditions, the experimenter closed the curtain in front of the competitor during the baiting of one of the cups and the lifting of the empty cup. This made that the dogs had knowledge about the content of 3 cups and the competitor of just one. Since the dog and competitor were sitting opposite each other, the dog could witness the competitor observing the baiting of only 1 cup. The first choice of the trial was always hidden to the opponent with the help of the curtains. The opponent would not see the choice, nor the handing of the treat to the chooser. The curtain would be reopened once the board was back in the middle. An example of a trial would thus look as followed: experimenter (E) closes the curtain in front of the competitor (C) → E lifts first cup, showing the dog subject (S) it remained empty → E opens curtain in front of C, lifts second cup and shows S and C how she hid a food reward under that cup → E closes curtain in front of C → E lifts third cup and shows S how she hid a food reward under that cup as well → E either leaves the curtain in front of C closed and rolls the board over to S for the first choice (SF), or opens the curtain in front of C, closes the curtain in front of S and rolls the board over to the competitor to make the first choice (CF) → S or C makes their choice → E lifts the chosen cup and hands the treat underneath, if any, to the chooser, then puts cup back → E rolls back the board to the middle and opens the curtain of the opponent, before rolling the board to that side. After the first choice was made, both curtains remained open for that trial.

In addition to the experimental conditions, there were two control conditions, each split into competitor first choice (CF) and subject first choice (SF) as well: All Known (AK-CF and AK-SF) and Competitor Ignorant (CI-CF and CI-SF). During the **All Known control condition**, the curtains in front of both individuals stayed open, and the dog witnessed how the competitor saw the baiting of both cups and lifting of the empty cup. This made that both dog and competitor had knowledge of the contents of all three cups. In AK-CF this also meant that the dog could see the first choice of the competitor, including the removal of the food reward from underneath the cup. This control checked if dogs followed the general course of events, as they should now avoid the cup chosen by the competitor. An example of a trial would thus look as followed: E lifts the first cup, showing both S and C how she hid a food reward underneath it → E lifts the second cup, showing both S and C how it remained empty → E lifts the third cup, showing both S and C how she hid a food reward underneath → E either rolls the board over to S for the first choice (SF), or to C for the first choice (CF) → S or C makes their choice → E lifts the chosen cup and hands the treat underneath, if any, to the chooser, then puts cup back → E rolls back the board to the middle, before rolling the board to the opponent’s side.

During the **Competitor Ignorant control condition**, the curtains in front of the competitor stayed closed, and the dog witnessed how the competitor saw none of the baiting. Just as in the experimental conditions, the first choice of the competitor, including the removal of the food reward, was invisible to the opponent. This checked if dogs exploited uncontrolled cues to solve the problem, and they should now choose randomly between the baited cups, even if the competitor chose first. An example of a trial would thus look as followed: E closes the curtain in front of C → E lifts the first cup, showing S how she hid a food reward underneath it → E lifts the second cup, showing S how she hid a food reward underneath it → E lifts the third cup, showing S that it remained empty → E either leaves the curtain in front of C closed and rolls the board over to S for the first choice (SF), or opens the curtain in front of C, closes the curtain in front of S and rolls the board over to C to make the first choice (CF) → S or C makes their choice → E lifts the chosen cup and hands the treat underneath, if any, to the chooser, then puts cup back → E rolls back the board to the middle and opens the curtain of the opponent, before rolling the board to that side. After the first choice was made, both curtains remained open for that trial.

Dogs participated in 24 experimental trials and 24 control trials divided over 2 days, each with its own starting condition (CF or SF). This resulted in 12 experimental trials and 12 control trials (6 CI, 6 AK) on a given day. Half of the dogs started with the CF condition, and half with the SF condition. The order of the trials (Exp, AK or CI) within each condition was randomized over the 24 trials on that same day, while making sure that a same set of cups was never baited two times in a row.

### Data processing and statistical analyses

The dogs’ choices for a specific cup in the 3-choice 2-reward set-ups were coded from videos by the experimenter. A second coder who was not involved in conducting the experiments recoded 20% of the trials, revealing high inter-observer reliability (Cohens Kappa = 0.98, *n* = 240). The choices were labelled as ‘Subject-only’, being the cup baited in view of only the dog; ‘Both’, being the cup baited in view of both; and ‘Empty’.

For each experimental condition, the 12 trials (choices) per dog were pooled and expressed as percentages for choosing cup Subject-only, Both or Empty, which were compared to chance (33.3%) using one sided t-tests. Similarly, for each control conditions, the 6 choices per starting condition (CF or SF) were pooled and in CF conditions expressed as percentages for choosing the cup chosen by the competitor, the cup not chosen by the competitor, or the Empty cup, and in SF conditions as percentages for choosing a baited or Empty cup. These choices were also compared to chance (33.3%) using one sided t-tests. Choices for cups were compared to each other using ANOVA’s, to see if one cup was chosen more or less than the other cups. Comparisons between conditions were made using paired t-tests. To test for a learning effect over days and an effect of starting condition, we fitted a linear mixed-effects model (LMM) with choice percentage as the response variable. Experimental day (1 or 2), starting condition (SF vs. CF), and cup type (Subject-Only, Both, Empty) and their interactions were included as fixed effects. Dog identity was included as a random intercept to account for repeated measures within individuals. The threshold for significance was 0.05 in all the tests. The RStudio (RStudio team 2024) script can be found back in the supplementary data (S5).

## Results

Twenty-two dogs competed over food with a human competitor in a 2-reward 3-choice set-up, by choosing 1 of 3 cups that were baited in view of only the dog (cup Subject-only), baited in view of both the competitor and the dog (cup Both) or left empty (cup Empty). Average (± stdev) choice percentages across the experimental conditions only, so based on 24 trials per dog (*n* = 22), were 42 ± 13% for the cup Subject-only, 36 ± 9% for Both and 22 ± 8% for Empty.

**Fig. 2:**
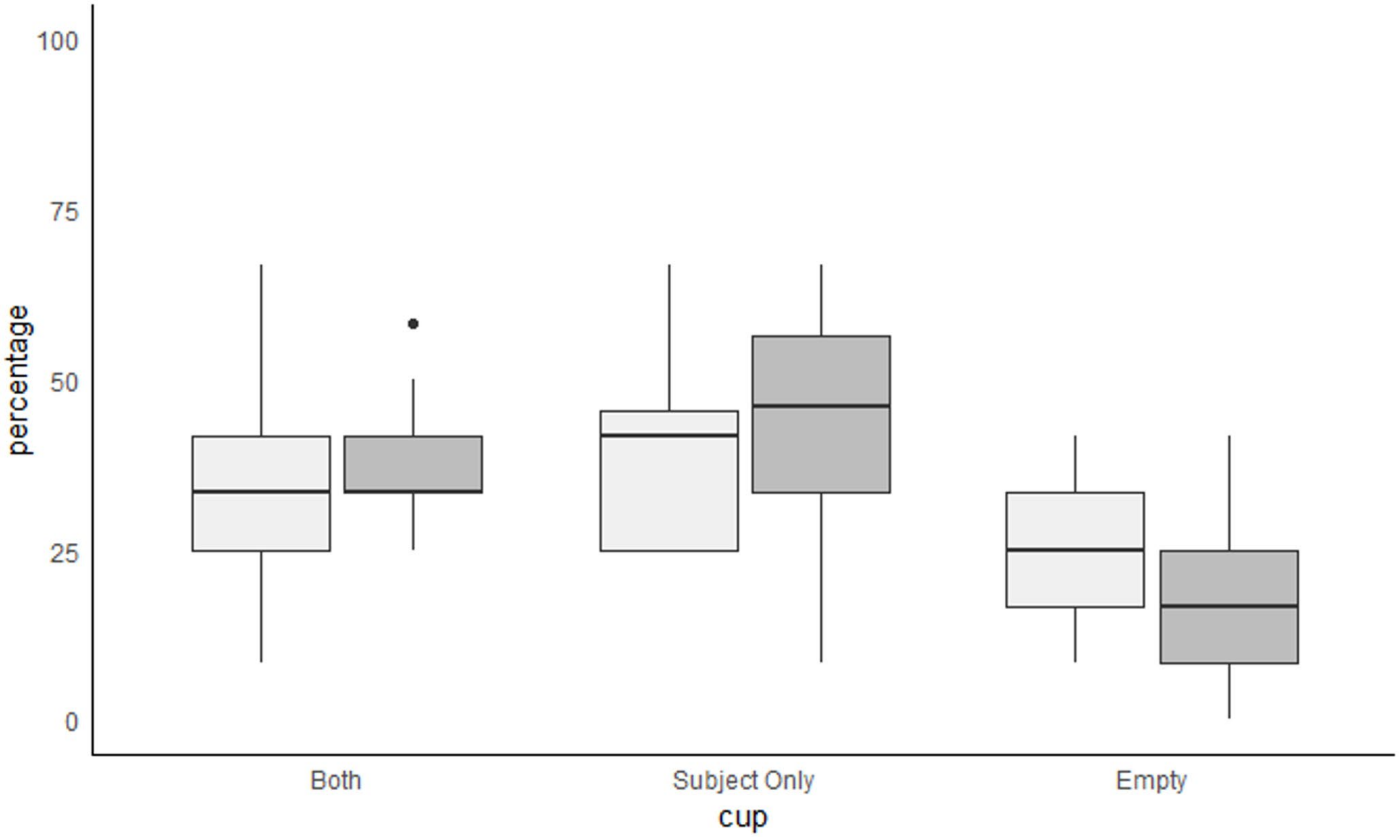
Choice percentages across 12 experimental trials per dog. Dogs (N = 22) competed over food with a human in a 3-choice food competition set-up, where the dog saw 2 cups being baited (cups Both and Subject-only) and the human competitor only 1 (Both). One cup remained empty (Empty). First choice was alternated between the dog subject (condition SF, light boxes) and competitor (CF, dark boxes)

The first choice during the experimental condition trials alternated between the dog subject (condition SF), after which dogs were expected to randomly choose between the baited cups Subject-only and Both, and the competitor (CF), after which dogs should choose Subject-only, and it was always hidden from the other individual. In the experimental condition SF, the dogs chose the Subject-only cup at chance level (38.05 ± 16.07%; t-test, df = 20, t = 1.378, *p* = 0.183), just as the Both cup (33.47 ± 15.23%; t = 0.043, *p* = 0.966; Fig. 2). The Empty cup was chosen below chance (23.93 ± 11.26%; t = −3.916, *p* = 0.001; Fig. 2). Cup preference differed (ANOVA, F = 4.951, *p* = 0.012), with post-hoc analysis showing a significant difference between choices for the Subject only and the Empty cup (t = −3.084, *p* = 0.010). There was no difference between choices for Both cup and the Empty cup (t = −2.084; *p* = 0.106), or between choices for the Subject only cup and the Both cup (t = −1.000, *p* = 0.581), showing that the dogs chose randomly between baited cups (S2 table).

In the experimental CF condition, where the human competitor chose first after having observed cup Both being baited, dogs were expected to avoid this cup and favour cup Subject-only. However, the outcomes did not support this and the dogs chose randomly between the baited cups. There was a statistical trend for dogs choosing the Both cup above chance (37.12 ± 9.87%; t-test, df = 21, t = 1.802, *p* = 0.086; Fig. 2), while the Subject-only cup was chosen above chance (43.94 ± 16.10%; t = 3.091, *p* = 0.006) and the Empty cup below chance (18.94 ± 11.26%; t=−5.993, *p* < 0.001; Fig. 2). Comparison between cups undescribed this difference (ANOVA, F = 15.2, *p* < 0.001), with significant differences between the Empty and Subject only cup (t=−5.333, *p* < 0.001) and the Empty and Both cup (t=−3.879, *p* = 0.001), where the baited cups were chosen more often than the Empty cup. The Both cup and the Subject only cup did not significantly differ (t=−1.455, *p* = 0.323; S2 table).

The dogs’ understanding about the human competitor’s knowledge was expected to shift their choices towards toward the Subject-only cup in the condition CF, but not SF. However, there was no significant difference between experimental conditions (CF, SF) for choosing the Subject-Only cup (paired t-test, t=−1.174, df = 20, *p* = 0.254). There was a trend for choosing the Empty cup more in the SF condition (t = 1.961, df = 20, *p* = 0.064), suggesting that dogs might have chosen more randomly when they chose first, including choosing the Empty cup more often.

The All Known (AK) control condition allowed both the dog and competitor to see the baiting of 2 cups, the first choice of the competitor and the reward delivery. This checked if dogs followed and understood the general course of events, by preferring the baited cup that the competitor did not choose. Dogs indeed showed a preference for the cup not chosen by the competitor (50.00 ± 21.21%) over the cup chosen by the competitor (30.30 ± 17.55%) when the competitor chose first (paired t-test, t = 2.603, df = 21, *p* = 0.017; S3 table). When the dog chose first, in the SF condition, dogs showed an expected preference for a baited cup (80.41 ± 23.39%) over the Empty cup (15.04 ± 15.39%; paired t-test, t = 9.201, df = 21, *p* < 0.001).

The Competitor-ignorant (CI) control condition allowed only the dog to see the baiting of the cups, and the competitor’s choice was hidden from the dogs. This checked if dogs acted upon unintended cues from the competitor, since they should have no reason to prefer one cup over the other, regardless of the conditions CF or SF. As expected, dogs did not show a preference and chose randomly between baited cups. They did surprisingly choose the baited cup not chosen by the competitor above chance in CF (51.52 ± 16.19%; t-test, df = 21, t = 5.268, *p* < 0.001; S4 table) and tended to choose the cup chosen by the competitor below chance (25.76 ± 18.35%; df = 21, t = −1.936, *p* = 0.066). However, a direct comparison of the choices for cup types revealed only a significant difference between the cup not chosen by the competitor and the Empty cup (22.73 ± 12.11%; t = −3.213, *p* = 0.006) and not between the cup chosen by the competitor and the cup not chosen by the competitor (t=−1.991, *p* = 0.153), showing no clear preference for one of the baited cups. In the SF condition, dogs showed the expected preference for a baited cup (75.00 ± 23.43%) over the Empty one (20.45 ± 17.01%; paired t-test, t = 7.320, df = 21, *p* < 0.001).

Dogs started with either a full day of SF trials or a full day of CF trials, which alternated the second testing day. To examine potential learning effects, the distribution of choices was compared between experimental days (Day 1 vs. Day 2), separately for each cup type and starting condition (CF vs. SF). There was no evidence for a learning effect across days or due to starting condition. There was no difference in choice percentage between experimental days for the Subject-Only (LMM, *p* = 0.183), Both (*p* = 0.092) or Empty (*p* = 0.721) cup, suggesting that there was no learning effect over the two days. There was also no main effect of starting condition (LMM, *p* = 1), indicating that dogs who began with SF did not differ overall in choice proportions from those who began with CF. Moreover, an insignificant interaction between starting condition and cup choice (LMM, *p* = 0.281), showed that cup choice did not depend on the condition the dog started with (CF or SF).

## Discussion

Domestic dogs have a good understanding of another’s visual perspective (e.g. Catala et al., 2017; Kaminski et al., 2009; Maginnity & Grace, 2014), but this does not mean that they therefore understand what a person logically knows based on what he/she saw earlier. Our dog subjects could earn food rewards by predicting the actions of a human female competitor in a competitive 3-choice 2-reward set-up that we adapted from Kaminski, Call and Tomasello (2008). If the experimental competitor chose first (Exp-CF), dog subjects chose above chance for the ‘Subject-only’ cup, that only they had seen being baited and thus logically should still have held a food reward. This gives the impression that our dogs understood what the competitor had seen and likely had chosen on the basis of what she had seen. However, they did not choose the Subject-only cup significantly more than the ‘Both’ cup, which both the dog and competitor had seen being baited and that would logically be emptied by the competitor in this CF condition. When the dog chose first (Exp-SF), dogs chose randomly between baited cups, as was expected. There was no significant condition effect (SF versus CF) on choice percentages for the different cups, meaning that the dogs did not account for who chose first. There was no difference between the two different testing days and there was no influence of the condition the dogs started with (SF or CF) on their choices, indicating no learning effects. Our dogs avoided the ‘Empty’ cup across the different conditions, as well as the cup that they saw being chosen by the competitor in the ‘All Known’ (AK) control condition. This indicates that they did understand the basic working principle of the study. However, they did not demonstrate an understanding of the competitor’s knowledge that came from what she had seen earlier.

The dogs seemed to follow the general course of events in this study, as they avoided the empty cup in general, as well as the cup that they saw being chosen by the competitor when the baiting of the cups was visible for all (during AK-CF). The task proved challenging though, since there was a considerable number of avoidable mistakes, like that a good quarter of the choices still went to the cup that the competitor chose in view of the dog. This could be explained by local enhancement or stimulus enhancement. Dogs are extremely attentive to human gestures, motivated to cooperate with humans, and attracted to places (local enhancement) or objects (stimulus enhancement) that they saw being visited or handled by humans (Hare & Tomasello, 2005; Ostojić & Clayton, 2014; Soproni et al., 2001). The handling of a cup by a human made dogs prefer it over one that they knew to hold a bigger food reward (Marshall-Pescini et al., 2012). Likewise, dogs preferred a bowl that was pointed at by an experimenter over one with olfactory or visual cues of a hidden food reward. Moreover, they were as likely to choose a bucket they saw being baited as one they were shown to be empty, but to which an experimenter was pointing (Szetei et al., 2003). This even happened when the reward holding containers were transparent and dogs could see that the experimenter was pointing at an empty container (Dwyer & Cole, 2018). This means that some of our dogs may have interpreted the pointing of the competitor at her time of choosing in the AK condition as a cooperative informative gesture, a form of stimulus enhancement. Mechanisms of local enhancement and stimulus enhancement may have influenced some dogs’ decision making, causing variation in choices and some unexpected outcomes. Alternatively, the motivation of some dogs to obey may override their motivation to get a food reward, even if they detect ‘wrong’ instructions from humans. Extensive control conditions should help to better understand what makes dogs decide in complex study designs like the present one.

Some dogs might have used uncontrolled cues to success in the task. In the Competitor Ignorant (CI) control condition, in which the competitor saw no baiting, the dogs showed no clear preference for either baited cup. However, they did choose only the baited cup that the competitor had not chosen above chance in the CI-CF condition, even though the competitor was blind to the baiting and made her choice out of sight of the dog. This suggests that some dogs might have used uncontrolled cues, which means that the choices for the non-chosen ‘Subject-only’ cup in the experimental CF condition may have been in part been due to uncontrolled cues. Uncontrolled cues could have been auditory as the curtains were not soundproof. The experimenter handled the cups and the food reward silently, but some dogs may have been able to hear which cup was lifted by the experimenter. Future studies could prevent this by having the experimenter lift all the cups after the choice. Unintentional (micro)movements by the competitor after the curtain was opened again might also have influenced the choice of the dogs. Dogs are apt in picking up subtle signals and changes in posture (McKinley & Sambrook, 2000), like an unintended glance in the direction of the chosen cup, which would have allowed our dogs to identify the filled cup by exclusion. To prevent this, all the choices could have been made blind. A Clever Hans effect by unintentional owner cues seems unlikely as dog owners were as blind to the first choice of the competitor as the dog was. Moreover, despite some dogs possibly using unintended cues, dogs did not choose the cup not chosen more often than the cup chosen by the competitor, nor did they pick the Subject-Only cup more often than the Both cup during the experimental CF trials. Therefore, the use of unintended cues in this study seems to be minimal.

The dogs’ lack of avoidance of the Both cup in the experimental Competitor First Choice (Exp-CF) condition, makes that we cannot conclude from this paradigm whether dogs understand human knowledge. If the dogs understood the task and would also have taken the knowledge of the competitor into account, they would have understood that the competitor only knew of the food reward in the Both cup. The competitor would therefore logically have chosen that cup, resulting in a shift in preference towards the Subject-only cup for the dogs in this condition. Instead, dogs did not prefer the Subject-only cup in the CF situation, nor was there evidence for a shift in preference over time. Nonetheless, the dogs did seem to understand the competitor’s action of choosing, as shown by the results from the AK condition. It cannot be excluded that some individual dogs understood the task. Even though the Subject-only cup was not chosen significantly more than the cup that both had seen baited, it was chosen above chance only in the condition that the competitor chose first, suggesting that some dogs did prefer this cup specifically in the Exp-CF condition. Dogs show considerable individual variation in several social cognitive tasks, reflecting factors like breed (Junttila et al., 2022) and obedience (Junttila et al., 2024). In our study, there seems to be variation in dogs’ responses, but the dataset is too small to take these individual factors into account. What factors make that some dogs understand cognitively more advanced tasks while most dogs do not, thus remains to be studied.

Our study design was based on the set-up previously designed for chimpanzees by Kaminski et al., (2008). In that study it was found that chimpanzees, like adult and infant humans, take the knowledge of the competing chimpanzee into account. They chose the cup only they had seen the baiting of above chance, when they were second to choose and more so than in the scenario where they chose first, showing an understanding that that cup would still hold a reward after their competitor had chosen (Kaminski et al., 2008). In our study we also found that dogs had no preference for a specific cup when they chose first, and that they chose the cup only they had seen the baiting of above chance when they were second to choose. In contrast with the chimpanzees and the humans, dogs showed no overall difference in their choice based on who chose first. In a comparison between cups, they seemed to also choose randomly between cups when the competitor chose first, suggesting that they did not take the knowledge of the competitor into account. This means that chimpanzees here seem to outperform dogs. The same study also found that adult and infant humans would prefer the cup both had seen being baited when they chose first, to maximise their final outcome. Both dogs and chimpanzees did not do this. It is likely that both species lack the strategic planning to take a possible second choice into account, as this requires the ability to calculate probabilities to some extent (Kaminski et al., 2008). A recent review argued that dogs seem to outperform chimpanzees on tasks that require perspective taking or deception, but that the kind of tasks in which they do so (e.g. social, cognitive, causal) seems to differ (Lea & Osthaus, 2018). In our study, the task at hand was competitive and likely better suited for chimpanzees than for dogs, which are used to cooperate with humans (Bray et al., 2021).

We chose to test knowledge attribution in a competitive setting, since dogs are a competitive species. Competition between pack living dogs can be seen around feeding, both in free ranging dogs (Berghänel et al., 2025) and in human-raised pack living dogs (Dale et al., 2017). However, the dogs used in this study were pet dogs which for generations have lived in human households where they were unlikely to experience competition in the same way as pack living dogs do. Nonetheless, pet dogs may show resource guarding towards humans, which could be a sign of the dogs seeing the human as competition (Jacobs et al., 2018). However, dogs have been shown to show increased deference towards humans (Range et al., 2019). In a study in which similarly pack-living and human-raised dogs and wolves had to cooperate with a human in a string-pulling task, both species were successful, but did show differences in their approach towards the human. While wolves lead, treating the human as an equal partner, dogs more often cooperated in a more deferential manner and waited for the human to lead. The authors concluded that dogs might have been selected for increased deference towards humans during domestication, to minimize conflicts over resources and ensure safe co-habitation and co-working (Range et al., 2019). This increased deference might have made it unlikely for some dogs to engage in competitive interactions with a human competitor. If the dogs did not see the competitor as competition, or did not want to compete with her, this may explain our findings that dogs did not take the choice of the competitor into account. The study set-up might work with two dogs, as resource guarding is evident also among pet dogs (Jacobs et al., 2018) and there is no indication of increased tolerance among dogs (Range & Virányi, 2014).

Research findings on knowledge attribution towards humans in dogs become more conflicting when dogs have to translate earlier visual perspective into present actions (Kaminski et al., 2009). A key experimental factor across several studies on the topic may be whether or not an experimenter keeps his/her eyes on the target. Dogs did well in knowledge-ignorance studies where the knowledgeable person stayed in the room with view on the target (e.g. Catala et al., 2017; Maginnity & Grace, 2014), but not in studies where the visual perspective of the knower was interrupted by walking out of the room or having a curtain closed in front of them (e.g. the current study and the one by Kaminski et al., (2008). In the former studies, dogs could track the knowledgeable person, including his/her gestures and gazes, whereas in the latter they had to recall his/her visual perspective and infer associated knowledge. It seems unlikely that dogs struggle with briefly remembering a human’s visual perspective or a reward location, as they can remember the location of an object that they saw being hidden for up to 240 s at least (Fiset et al., 2003). However, complex studies such as this one and the one by Kaminski et al., (2009) might involve too many cognitive steps or too many distracting actions, including movement of the person and pointing gestures, which might hamper dogs in showing an understanding of human knowledge. One thing that might reduce the amount of cognitive steps and improve the dogs’ performance is giving the dogs more direct experience with the set-up, like placing the dogs in the position of not seeing the baiting, like the competitor.

The several cognitive steps required in the current study included an understanding of visual perspective and object permanence, and the inference of how the observation of a baiting translated into an obvious choice by the competitor. Dogs are clearly skilled in following a person’s gaze and taking their present visual perspective (e.g. Gácsi et al., 2004; Kaminski et al., 2009; Soproni et al., 2001). Understanding object permanence, in that food rewards still exist when covered by a cup, should neither be a major obstacle for most dogs. Dogs readily master visible displacement tasks and understand that an object continues to exist after it disappears into a container (Fiset & Plourde, 2013; Zentall & Pattison, 2016). The inference of causality between relevant events may have posed a more serious challenge. In string-pulling tasks, dogs showed no instantaneous understanding of means-end connections (Osthaus et al., 2005; Range et al., 2012). When dogs had to locate hidden food with the help of either physical causal cues, like food in a cup making noise when shaken, or human communicative cues, like pointing, they were skillful in using human cues and not in inferring causality (Bräuer et al., 2006). In this, the type of task matters (Riemer et al., 2014), since dogs did successfully solve an “on/off” task (Range et al., 2011). This task is based on the ‘support problem’ (Piaget & Cook, 1952), and dogs had to distinguish between a board with a reward on it, and one with the reward next to it. They showed this distinction even when one of the rewards was placed closer to the dog, indicating that dogs overcame the proximity bias and solved this means-end tasks (Range et al., 2011). Therefore, despite the set task requiring a cumulation of cognitive abilities, we assumed it doable for dogs given earlier findings on the separate task aspects, but the combination may have been too demanding.

The complexity of the task at hand becomes already evident when looking at the high dropout rate already during training. Of the 42 dogs that participated in the training, only 24 passed it. This means that almost half of the participating dogs dropped out during various moments in training. This could either be in the early training rounds, if they refused to choose, or kept choosing randomly, or during the pretest, if they developed a side preference, or were negatively influenced by the actions of the competitor. Dogs that refused to choose or kept choosing randomly were likely stressed, not food motivated enough, too impulsive (always going for the cup straight ahead) or too heavily trained in searching for food. Many dog owners play food-related searching games, in which the dog has to systematically search for food rewards. We found that some of the dogs in the same way would systematically investigate the cups, not taking into account what they had witnessed. Since the first cup indicated would count as the choice, these dogs would drop out of the training early. Dogs that passed the training rounds but dropped out during the pretest often developed a side preference that could not be trained out within two training days that were planned for each dog. This sudden side preference may have been a result of the dogs being confused or frustrated by the inclusion of the competitor, who would also choose one of the baited cups and take one food reward. We noticed some of the dogs also exclusively choosing the cup previously chosen, in sight, by the competitor. So, local enhancement or stimulus enhancement, which have been discussed before, might also have played a role in the high dropout rate. We chose to only continue with the dogs that made the pretest within the two training days, which each included 4 pre-test sessions of 6 trials each. By doing so, we might have introduced a selection bias towards highly (food) motivated and compliant dogs.

We were unable to demonstrate with our set-up that dogs in general understand knowledge that comes from earlier visual access in humans. However, it is uncertain at what level of the complex task within our study dogs failed. They may have struggled to understand the causality between the person’s earlier observation and present action, or may have been misdirected by simple mechanisms like local enhancement that often reflect dogs’ sensitivity to human cues. Moreover, the competitive paradigm might not work with pet dogs and humans due to dogs increased deference. The present lack of evidence for knowledge attribution in dogs does not mean that dogs do not have it, but our complex experimental set-up may have prevented them from showing it. Dogs’ dependence on humans makes it plausible for them to have a certain understanding of humans, including their knowledge; and more alternative methods of testing canine social cognition should be refined and tested. A better understanding of dog’s social cognition, and the factors that underlie individual differences, allow for improving the husbandry and training methods for pet dogs, ultimately improving the welfare of privately owned dogs.

## Supplementary Information

Below is the link to the electronic supplementary material.

Supplementary Material 1

Supplementary Material 2

Supplementary Material 3

Supplementary Material 4

Supplementary Material 5

## Data Availability

Raw data is provided in the supplementary material of this article and processed data is made available on Pure.
