# Supplementary material for: Do dogs know what humans know? A study into pet dogs’ (Canis familiaris) ability to attribute knowledge to an unfamiliar person
Source: Anim Cogn. 2025 Dec 12;29(1):10. doi: 10.1007/s10071-025-02034-0 (PMC12799639; doi:10.1007/s10071-025-02034-0)
Supplement: Supplementary file 1 — Supplementary Material 1 [file 10071_2025_2034_MOESM1_ESM.docx]

| **Name** | **Sex** | **Age (years)** | **Breed** |
| --- | --- | --- | --- |
| Sully | Male | 5 | Dachshund |
| Mike | Male | 5 | Dachshund |
| Sailor | Male | 6 | Irish water spaniel |
| Doodie | Female | 6 | Border terrier |
| Lucca | Male | 6 | Labrador retriever |
| Caspar | Male | 5 | Whippet |
| Bertie | Male | 4 | Cavalier king Charles spaniel |
| Ozzi | Male | 4 | Labrador retriever |
| Monty | Male | 4 | Labrador retriever |
| Vialli | Male | 5 | Whippet |
| Phoenix | Male | 5 | Whippet |
| Fearne | Female | 7 | Cocker spaniel |
| Fillipa | Female | 3 | Labrador retriever |
| Brooke | Female | 3 | Cocker spaniel |
| Shadow | Female | 10 | Border collie |
| Marj | Female | 9 | Irish water spaniel |
| Rocket | Male | 5 | Whippet |
| Pippa | Female | 10 | West highland terrier |
| Mali | Female | 3 | Sussex spaniel |
| Rafi | Male | 1 | Jack Russel terrier × Springer spaniel |
| Digby | Male | 3 | Border terrier |
| Rue | Female | 3 | Labrador retriever |
