# Supplementary material for: Do dogs know what humans know? A study into pet dogs’ (Canis familiaris) ability to attribute knowledge to an unfamiliar person
Source: Anim Cogn. 2025 Dec 12;29(1):10. doi: 10.1007/s10071-025-02034-0 (PMC12799639; doi:10.1007/s10071-025-02034-0)
Supplement: Supplementary file 4 — Supplementary Material 4 [file 10071_2025_2034_MOESM4_ESM.docx]

| **Name** | **Condition** | **Starting condition** | **Count successful** | **Count unsuccessful** | **Percentage successful (%)** |
| --- | --- | --- | --- | --- | --- |
| Sully | SF | SF | 6 | 0 | 100 |
| Sully | CF | SF | 3 | 3 | 50 |
| Mike | SF | SF | 6 | 0 | 100 |
| Mike | CF | SF | 3 | 3 | 50 |
| Bertie | SF | SF | 4 | 2 | 66.67 |
| Bertie | CF | SF | 2 | 4 | 33.33 |
| Brooke | SF | SF | 6 | 0 | 100 |
| Brooke | CF | SF | 4 | 2 | 66.67 |
| Caspar | SF | CF | 5 | 1 | 83.33 |
| Caspar | CF | CF | 1 | 5 | 16.67 |
| Digby | SF | CF | 5 | 1 | 83.33 |
| Digby | CF | CF | 3 | 3 | 50 |
| Doodie | SF | SF | 5 | 1 | 83.33 |
| Doodie | CF | SF | 1 | 5 | 16.67 |
| Fearne | SF | SF | 4 | 2 | 66.67 |
| Fearne | CF | SF | 1 | 5 | 16.67 |
| Lucca | SF | CF | 5 | 1 | 83.33 |
| Lucca | CF | CF | 3 | 3 | 50 |
| Mali | SF | CF | 5 | 1 | 83.33 |
| Mali | CF | CF | 2 | 4 | 33.33 |
| Marj | SF | SF | 5 | 1 | 83.33 |
| Marj | CF | SF | 4 | 2 | 66.67 |
| Monty | SF | CF | 6 | 0 | 100 |
| Monty | CF | CF | 4 | 2 | 66.67 |
| Ozzi | SF | SF | 4 | 2 | 66.67 |
| Ozzi | CF | SF | 2 | 4 | 33.33 |
| Phoenix | SF | SF | 3 | 3 | 50 |
| Phoenix | CF | SF | 2 | 4 | 33.33 |
| Pippa | SF | SF | 6 | 0 | 100 |
| Pippa | CF | SF | 5 | 1 | 83.33 |
| Rafi | SF | SF | 6 | 0 | 100 |
| Rafi | CF | SF | 3 | 3 | 50 |
| Rocket | SF | CF | 4 | 2 | 66.67 |
| Rocket | CF | CF | 3 | 3 | 50 |
| Rue | SF | CF | 6 | 0 | 100 |
| Rue | CF | CF | 2 | 4 | 33.33 |
| Sailor | SF | SF | 4 | 2 | 66.67 |
| Sailor | CF | SF | 5 | 1 | 83.33 |
| Shadow | SF | CF | 6 | 0 | 100 |
| Shadow | CF | CF | 4 | 2 | 66.67 |
| Vialli | SF | CF | 5 | 1 | 83.33 |
| Vialli | CF | CF | 4 | 2 | 66.67 |
| Fillipa | CF | CF | 5 | 1 | 83.33 |
