# Supplementary material for: Do dogs know what humans know? A study into pet dogs’ (Canis familiaris) ability to attribute knowledge to an unfamiliar person
Source: Anim Cogn. 2025 Dec 12;29(1):10. doi: 10.1007/s10071-025-02034-0 (PMC12799639; doi:10.1007/s10071-025-02034-0)
Supplement: Supplementary file 5 — Supplementary Material 5 [file 10071_2025_2034_MOESM5_ESM.docx]

library(readxl)

library(extrafont)

library("tidyverse")

library("here")

library("patchwork")

library("knitr")

library("kableExtra")

library("gt")

library("gtsummary")

library("broom")

library("tinytable")

library("ggplot2")

library('lme4')

library(lmerTest)

library(“reshape2”)

library(“tidyr”)

library(“dplyr”)

library("ez")

library(“emmeans”)

data <- read_excel("data UK.xlsx")

data_controls <- read_excel("data UK_complete.xlsx")

data2 <- melt(data, id.vars = "FourFactor")

data_long <- gather(data, cup, percentage, SeenPerc:EmptyPerc, factor_key=TRUE)

treatment <- as.factor(data$FourFactor)

data$Dog_Name <- factor(data$Dog_Name)

##Descriptives ####

long_data <- data_controls %>%

pivot_longer(

cols = c(Both_pct, Subject_only_pct, Empty_pct),

names_to = "Cup",

values_to = "Percentage"

)

summary_stats <- long_data %>%

group_by(Condition, Treatment, Cup) %>%

summarise(

mean_pct = mean(Percentage),

sd_pct = sd(Percentage),

n = n(),

.groups = "drop"

)

## Plots ####

ggplot(data = data_long, aes(x=cup, y=percentage)) + geom_boxplot(aes(fill=FourFactor))

p <- ggplot(data = data_long, aes(x = cup, y = percentage)) +

geom_boxplot(aes(fill = FourFactor)) +

ylim(0, 100) +

scale_x_discrete(labels = c("SeenPerc" = "Both", "UnseenPerc" = "Subject Only", "EmptyPerc" = "Empty")) +

theme_minimal() +

theme(panel.grid = element_blank(), axis.line = element_line(), legend.position = "none")

p

p+scale_fill_brewer(palette="Greys")

## Paired t-test ####

df_wide <- data %>%

select(Dog_Name, FourFactor, UnseenPerc) %>%

pivot_wider(names_from = FourFactor, values_from = UnseenPerc) %>%

drop_na(DF, HF) # Remove dogs missing HF or DF

t_test_result <- t.test(df_wide$DF, df_wide$HF, paired = TRUE)

print(t_test_result)

df_wide_empty <- data %>%

dplyr::select(Dog_Name, FourFactor, EmptyPerc) %>% # Use EmptyPerc instead of UnseenPerc

pivot_wider(names_from = FourFactor, values_from = EmptyPerc) %>%

drop_na(DF, HF) # Remove dogs missing HF or DF

t_test_empty <- t.test(df_wide_empty$DF, df_wide_empty$HF, paired = TRUE)

print(t_test_empty)

##t-tests against chance experimental ###

treatments <- c("DF", "HF")

cups <- c("UnseenPerc", "SeenPerc", "EmptyPerc")

for (treat in treatments) {

cat("\n=== Treatment:", treat, "===\n")

subset_data <- filter(data, FourFactor == treat)

for (cup in cups) {

test_result <- t.test(subset_data[[cup]], mu = 33.33)

cat("Cup:", cup, "\n")

print(test_result)

cat("\n")

}

}

## comparisons between cups ###

long_data <- data %>%

pivot_longer(cols = c(UnseenPerc, SeenPerc, EmptyPerc),

names_to = "Cup",

values_to = "ChoicePerc")

df_data <- filter(long_data, FourFactor == "DF")

hf_data <- filter(long_data, FourFactor == "HF")

# ANOVA

aov_result <- aov(ChoicePerc ~ Cup + Error(Dog_Name/Cup), data = df_data)

summary(aov_result)

emmeans(aov_result, pairwise ~ Cup)

aov_result_h <- aov(ChoicePerc ~ Cup + Error(Dog_Name/Cup), data = hf_data)

summary(aov_result_h)

emmeans(aov_result_h, pairwise ~ Cup)

## t-tests including control conditions ####

conditions <- unique(data_controls$Condition)

treatments <- unique(data_controls$Treatment)

cups <- c("Both_pct", "Subject_only_pct", "Empty_pct")

data_controls <- data_controls %>%

mutate(

Both_pct = Both_pct * 100,

Subject_only_pct = Subject_only_pct * 100,

Empty_pct = Empty_pct * 100

)

# t-tests

for (cond in conditions) {

for (treat in treatments) {

subset_data <- filter(data_controls, Condition == cond, Treatment == treat)

cat("\n--- Condition:", cond, "- Treatment:", treat, "---\n")

for (cup in cups) {

test <- t.test(subset_data[[cup]], mu = 33.33)

cat("Cup:", cup, "- t =", round(test$statistic, 3),

", p =", round(test$p.value, 4), "\n")

}

}

}

filter(data_controls, Condition == "AK", Treatment == "DF") %>%

summarise_all(sd)

#Test CI-HF

ci_hf <- data_controls %>% filter(Condition == "CI", Treatment == "HF")

ci_hf_long <- ci_hf %>%

select(Dog_ID, Both_pct, Subject_only_pct, Empty_pct) %>%

pivot_longer(cols = -Dog_ID, names_to = "Cup", values_to = "Percentage")

# ANOVA

ezANOVA(

data = ci_hf_long,

dv = Percentage,

wid = Dog_ID,

within = .(Cup),

type = 3,

detailed = TRUE

)

aov_ci_hf <- aov(Percentage ~ Cup + Error(Dog_ID/Cup), data = ci_hf_long)

emmeans(aov_ci_hf, pairwise ~ Cup, adjust = "bonferroni")

subject_only_vals <- ci_hf$Subject_only_pct

t.test(subject_only_vals, mu = 33.33)

both_vals <- ci_hf$Both_pct

t.test(both_vals, mu = 33.33)

#Test CI-DF

ci_df <- data_controls %>% filter(Condition == "CI", Treatment == "DF")

t.test(ci_df$Subject_only_pct, ci_df$Empty_pct, paired = TRUE)

#Test AK-HF

ak_hf <- data_controls %>% filter(Condition == "AK", Treatment == "HF")

t.test(ak_hf$Subject_only_pct, ak_hf$Both_pct, paired = TRUE)

ak_hf_long <- ak_hf %>%

select(Dog_ID, Both_pct, Subject_only_pct, Empty_pct) %>%

pivot_longer(cols = -Dog_ID, names_to = "Cup", values_to = "Percentage")

aov_ak_hf <- aov(Percentage ~ Cup + Error(Dog_ID/Cup), data = ak_hf_long)

emmeans(aov_ak_hf, pairwise ~ Cup, adjust = "bonferroni")

#Test AK-DF

ak_df <- data_controls %>% filter(Condition == "AK", Treatment == "DF")

t.test(ak_df$Subject_only_pct, ak_df$Empty_pct, paired = TRUE)

# between days ####

m <- lmer(

percentage ~ ExpDay * FourFactor * cup + (1 | Dog_Name),

data = data_long

)

anova(m)

summary(m)

library(emmeans)

emm <- emmeans(m, ~ ExpDay | cup)

pairs(emm) # compares Day 1 vs Day 2 for each cup

emm2 <- emmeans(m, ~ ExpDay * FourFactor | cup)

pairs(emm2) # day × condition differences

#effect of starting condition

data_long <- data_long %>%

group_by(Dog_Name) %>%

mutate(StartCond = first(FourFactor[ExpDay == 1])) %>%

ungroup()

l <- lmer(

percentage ~ ExpDay * cup * StartCond + (1 | Dog_Name),

data = data_long

)

anova(l)

ell <- emmeans(l, ~ ExpDay | cup)

pairs(ell)
